# Professional Courtesies in Connection with What Are Commonly Considered Failures in Dental Practice

**Published:** 1887-02

**Authors:** C. E. Francis

**Affiliations:** New York.


ARTICLE II.

### PROFESSIONAL COURTESIES IN CONNECTION WITH WHAT ARE COMMONLY CONSIDERED FAILURES IN DENTAL PRACTICE.

BY C. E. FRANCIS, D. D. S., NEW YORK.

The course of human effort seldom runs smooth, and the daily routine of life is checkered by varied experiences that denote a perpetual contest between fortune and fate.

An ancient proverb declares that "All creatures are victims to circumstances," and certain it is that to a large extent circumstances frame men's opinions and govern their actions. And likewise do circumstances revolutionize established ideas, modify fixed methods and vary the ever-flowing currents that sweep the shores of prosperity or adversity. Inclination may suggest enterprises which adverse circumstances render difficult or impossible to execute, and tasks undertaken with fair hopes of success often terminate in failures and disappointments. Unforeseen contingencies possibly arise; unfavorable conditions appear; opposing influences prevail to frustrate earnest efforts or disappoint reasonable expectations.

A dentist's experience is exceedingly varied and beset with numberless difficulties, perplexities and trials. He may be ever so skillful in the dental art and possess the requisites that especially fit him for his calling, yet he cannot invariably overcome impending obstacles, nor count on uniform or absolute success. No man is infallible, however he may boast, for infallibility is not a human attainment. Failures occur in all quarters, and every mortal has a proportionate share of them. If individuals see not their own imperfections, perhaps others do, yet occasional ill success does not indicate absence of manipulative ability, nor a total lack of talent or genius.

It is well to hesitate before condemning operations that our neighbors have performed, for it is possible that no better results would have followed had like tasks, under similar circumstances been attempted within our own doors. Before hurling offensive missiles at others, it may be wise to consider if the fortifications erected for our own shelter are not citadels of glass.

It is unfair to judge harshly from a failure observed, and to gauge its author accordingly, for perhaps other operations from the same source might exhibit evidences of marked skill and sound judgment. Unfortunate it is, that some individuals are too ready to pass unfavorable comment upon the works of others, especially if in the least degree faulty, without possessing sufficient generosity to credit them for achievements that are eminently successful, and that bear the stamp of excellence.

It would almost seem as if in every trade or profession certain members imagine that the only possible way of gaining a reputation for themselves is in undermining the reputations of other members of their calling. In efforts to reach the goal of fame they would make stepping stones of their fellows, and do them injury at every tread. Sensible people, however, are not unlikely to distrust those who speak uncharitably of their compeers, and it frequently happens that ungenerous comments reflect unfavorably upon parties who utter them.

When dismissing our patients, we are not always sure that they will return to us for future treatment. In the course of time many will get into other hands. Some remove to distant localities, and find it inconvenient or impossible to come again. Others, by nature, are inclined to wander and are fond of making changes. Some change with a view to economy; others, perhaps, from lack of confidence or a fancied neglect. Some fail to return because bills for former operations remain unpaid, and such parties are usually ready to misrepresent or malign those whom they have defrauded.

Many an excellent and faithful dentist has been declared the author of discreditable operations which he never performed. Many have been charged with having inserted fillings (with assertions that they soon after "fell out,") in cavities that had never experienced the touch of a dental instrument! The decay and destruction of entire dentures, resulting from sheer neglect and carelessness on the part of those who possess them, are often charged as mal-practice on the part of some dentist who, perhaps, simply introduced a single filling, or removed beds of calculus and polished the stained surfaces of enamel.

" Your dentist has shamefully neglected your teeth and allowed them to go to destruction;" remarked a New York dentist to a lady, who, in emergency, called upon him with a request that he would quiet the rebellious demonstrations of an aching bicuspid, which, however, he failed to accomplish. Could this man have known the history of the case, he probably would not have ventured on so untruthful a statement. Fortunately, the lady rebuked him for the unjust insinuation.

To take for granted all that comes to our ears from disaffected or grumbling visitors is neither wise nor just, and certainly "fuel should not be added to the fire" by sympathizing with their complaints or endorsing their scandal.

There are various ways of doing injustice and injury to our neighbors, even without charging them with incompetency or denouncing them as charlatans. A feigned look of astonishment when scrutinizing their work, a significant shrug of the shoulder, or a disapproving shake of the head, will have the effect of undoing confidence in the operations of their former dentists, and sometimes prove even more damaging than open denunciations. To ask if the doctor was not in a hurry when he filled their teeth—if the doctor *himself* performed the operations—if the work was not done by his student—if the doctor's eyesight is not failing him, etc., are insinuations that excite suspicion, and convey the idea that operations have been slighted. Nor does it make things smoother to add in a semi-apologetic manner that "the doctor was considered a pretty fair sort of a dentist *once*, but unfortunately he is getting old." All this is needless, and generally uncalled for. It inflicts injury on those to whom such references are made, and fills with distrust the minds of those who have received their attentions. And to sum up, no good whatever can result from such ungenerous criticisms.

The causes that tend to failures following dental operations are many, and when duly considered, it seems a wonder that there are not more of them. Very many individuals defer their visits to the dentist until driven by dire necessity to seek relief from pain, and it is then found that their teeth are in a sad plight. Some cases present large approximal cavities, or crowns so decalcified and broken down that reliable walls for the retaining of fillings can hardly be secured. Exposed pulps, congested pulps, dead pulps and alveolar abscesses, also manifest their presence; and yet it is often expected that such dilapidated and diseased organs can be so restored that they will promise even better than before they became so wretchedly neglected or abused.

People who are so wilfully careless and negligent concerning the preservation of their organs of mastication are not entitled to a very great degree of sympathy if trouble ensues. Some sufferers seem to obtain a little grim satisfaction if they can only saddle the responsibility of their mishaps upon others, and their dentist is, in some instances, a very convenient scapegoat on whom to work their saddle. When discontented parties come to us with their complaints, it is clearly our duty to vindicate the good standing of our *confreres* as the occasion offers, and at the same time remind our visitors that personal interest and vigilant care on their part is requisite, if they expect to escape consequences that neglect is apt to engender.

A few days ago a letter came to me from Dr. Quinby, of Liverpool, in reply to a communication previously addressed to him concerning one of his patients, who, when on a recent flying trip to this country, called on me for a little personal attention. The doctor, in his letter, pictured the patient to a "bird of passage," who never thinks of treating his teeth to any sort of attention until just ready to start for some distant clime, then allowing such limited time for treatment that they cannot be properly cared for. Dr. Quinby also adds: "There is no satisfaction in trying to do anything for men who do not take an interest in their teeth themselves—men who try to make a sort of a father-confessor of their dentist, getting absolution from him once a year, and throwing all their sins of omission and commission on his shoulders." Here, gentlemen, you have a comprehensive essay in a few words, and from a fellow practitioner whose utterances are noted for wisdom and sound sense—a gentleman who well understands the difficulties that his fraternity have to contend with, and who is ready to sustain and defend them at every point.

And now, fellow practitioners, let us do justice to others as we would wish justice done to ourselves, and may we never forget that professional courtesies of every sort are much like "bread cast upon the waters," rewarding us with happy reflections, and inducing a reciprocation of kindly courtesies, with the hearty good-will and esteem of our professional brothers.—*Independent Practitioner.*

